# Conserved cell populations in doxorubicin-resistant human nasal natural killer/T cell lymphoma cell line: super multidrug resistant cells?

**DOI:** 10.1186/s12935-018-0644-6

**Published:** 2018-10-01

**Authors:** Xudong Zhang, Xiaorui Fu, Meng Dong, Zhenzhen Yang, Shaoxuan Wu, Mijing Ma, Zhaoming Li, Xinhua Wang, Ling Li, Xin Li, Zhenchang Sun, Yu Chang, Feifei Nan, Jiaqin Yan, Yun Mao, Mingzhi Zhang, Qingjiang Chen

**Affiliations:** Department of Oncology, The First Affiliated Hospital of Zhengzhou University, Νo. 1 Jianshe East Road, Zhengzhou, 450052 Henan People’s Republic of China

**Keywords:** ENKL, SP cells, EBV, MDR, Cellular heterogeneity

## Abstract

**Background:**

Extranodal NK/T-cell lymphoma, nasal type (ENKL) is a distinct clinicopathological entity and EBV-associated disease that is highly aggressive. Many patients had failed to respond to conventional chemotherapy or relapsed after treatment. Multi-drug resistance is a major cause that leads to these desperate failures. However, the specific mechanism of drug resistance is still unclear.

**Methods:**

In the previous study, we firstly developed a doxorubicin-resistant ENKL cell line known as SNK-6/ADM, and then a small quantity of side population (SP) cells were derived from SNK-6/ADM and named SNK-6/ADM-SP. In order to explore the biological characteristics and mechanism of drug-resistance of these cells, SNK-6, SNK-6/ADM and SNK-6/ADM-SP cells were utilized to evaluate potentially differences of chemotherapy resistance index (RI), morphology, proliferation, cell cycles, expression of ATP-binding cassette (ABC) transporters (ABCG1, ABCG2 and ABCC4) and surface markers, cytokine sensitivity, and situation of EBV infection.

**Results:**

We identified SNK-6/ADM-SP is a specific multidrug resistant cell population with a higher level of RI than SNK-6/ADM. Relevant evaluations showed that SNK-6/ADM-SP presented a series of conserved biological behaviors including relatively poor proliferation ability, high expression of ABCG2, weak sensitivity to IL-15 which could stimulate normal ENKL cells’ proliferation and differentiation, and EBV inhibition with low level of EBV-DNA replication and EBV-antigen expression.

**Conclusions:**

This discovered cellular heterogeneity of ENKL could provide a new perspective to better understand the mechanisms of drug resistance and overcome elusive response to chemotherapy of ENKL.

## Background

Extranodal natural killer (NK)/T-cell lymphoma (ENKL) is a distinct subtype of non-Hodgkin’s lymphoma, with a geographical predilection for Asian population [1]. ENKL predominantly occurs in the nasal or paranasal areas, and exhibits aggressive clinical features, characterized primarily by local indeterminate growth and destructive invasion [2]. Epstein–Barr virus (EBV) is usually detected in ENKL cells and plays an important role in lymphomagenesis. In most ENKL cases, the disease leads to significant chemotherapy resistance and poor clinical outcomes, probably due to the expression of multidrug-resistant genes and close association with EBV infection [3, 4]. Treatment regimens containing l-asparaginase (l-ASP) or pegaspargase have elicited promising responses [5, 6]. Hematopoietic cell transplantation (HCT) and targeted therapy have shown potential for clinical application [7, 8]. However, the therapeutic effect and prognosis of ENKL is still relatively poor compared with other types of lymphoma, and recurrent or refractory phenomenon usually emerges in the process of treatment. The fundamental reasons include lack of insight into biological characteristics of ENKL, such as cytological heterogeneity, multidrug resistance, EBV infection/latency/reactivation, and more importantly, the interaction between different factors.

Side population (SP) cell sorting, initially used for the identification of primitive hematopoietic stem cells, has been applied to enrich stem cell compartments in diverse tissues and organs [9]. SP cells are determined by their efflux of the fluorescent dye, Hoechst 33342 through an adenosine triphosphate (ATP)-binding cassette (ABC) membrane transporter. To elucidate the drug resistance and heterogeneous characteristics of nasal NK/T-cell lymphoma cells, we developed a doxorubicin-resistant cell line of ENKL known as SNK-6/ADM and identified the SP cells. Surprisingly, these cells exhibited conserved biological features and stronger chemotherapy resistance suggesting that human ENKL cells comprise heterogeneous populations of malignant NK cells, which may play an important role in the treatment of nasal NK/T-cell lymphoma.

## Materials and methods

### Doxorubicin-resistant cell lines

The EBV-positive ENKL cell line, SNK-6, established from primary lesions with nasal NK-cell lymphoma [CD3ε+, CD20−, CD56+, CD3(Leu4)−, EBER(+)] [10], was kindly provided by Dr. Norio Shimizu and Yu Zhang of Chiba University. Cells were cultured in RPMI 1640 supplemented with 10% heat-inactivated human serum and 700 U/mL recombinant human interleukin 2, 2M glutamine, 100 IU/mL penicillin, and 100 g/mL streptomycin sulfate in a 5% CO_2_-containing atmosphere.

Resistance to doxorubicin (ADM) was induced in SNK-6 by gradually increasing the concentration of doxorubicin in the culture medium with 2 μg/mL initial concentration. The resistance index (RI) was evaluated as follows: RI = IC50 (SNK-6)/IC50 (SNK-6/ADM). SNK-6/ADM cells were cultured in a medium containing 6 μg/mL ADM or without ADM 48 h before experiments.

### Methylthiazole tetrazolium (MTT) assay

Cells (4 × 10^4^ per well) seeded in 96-well plates were cultured in 180 μL medium, supplemented with 20 μL of chemotherapeutic drug (doxorubicin, cytarabine, cisplatin, gemcitabine, l-asparaginasum) in each well with a concentration gradient for 48 h. MTT (20 μL, 5 mg/mL) was added to each well, and incubated for 4 h at 37 °C in a 5% CO_2_-containing atmosphere. The absorbance at 490 nm (A) was measured with a spectrophotometer. The inhibition ratio was measured as a percentage of untreated controls. Measurements were conducted in duplicate and experiments were repeated at least three times. Inhibition rate (%) = (Control OD value − Experiment OD value/Control OD value) × 100%. IC50 was finally calculated by linear regression method.

### Side population (SP) cells

SP cells of SNK-6 and SNK-6/ADM were sorted by Hoechst33342 assay with flow cytometry as described previously [11]. We designated SP cells of SNK-6/ADM as SNK-6/ADM-SP. The SP cells of SNK-6 were not sorted due to small sample size.

### Cell transfection

For transfection, SNK-6/ADM and SNK-6/ADM-SP cells were cultured at 50–70% confluency without antibiotics. The short-hairpin RNA plasmids directly knocking down ABCC4 (sh-ABCC4), ABCG2 (sh-ABCG2) or the non-targeting sequence (sh-control) were chemically synthesized by Sangon (Shanghai, China). Plasmid transfection into SNK-6/ADM-SP cells was performed using Lipofectamine 2000 (Invitrogen, Carlsbad, CA, USA) according to the manufacturer’s protocol.

### Western blot

Cell protein extracts were immunoblotted with a primary antibody followed by a secondary antibody. Primary antibodies (P-gp, ABCG2, ABCC4, LMP1) were used at a diluted concentration of 1:2000 while the secondary antibody was used at 1:10,000. Protein bands were visualized with enhanced chemiluminescence. Equal protein loading was confirmed with anti-β-actin antibody, which was diluted as recommended by the manufacturer.

### Cell cycle assay

Cells were collected, and fixed with 70% cold ethanol. The fixed cells were treated with 50 μg/mL of DNase-free RNase and 20 μg/mL of propidium iodide. A total of 10,000 cells were analyzed by FACScan. Cell cycle was detected, and cells in G0/G1, S, and G2/M phases were evaluated.

### Expression of surface markers

Cells were analyzed by two-color immunofluorescence using flow cytometry to determine the expression of surface markers. The following antibodies conjugated with fluorescein isothiocyanate (FITC) were used: anti-CD16, -CD25, -CD56, -CD34, -CD117, and -CD122.

### IL-15 sensitive assay

SNK-6, SNK-6/ADM and SNK-6/ADM-SP cells (4 × 10^4^ per well) were treated with 10 ng/mL IL-15 for 48 h, stained with 20 μL MTT (5 mg/mL) and subsequently dissolved in 100 μL of DMSO. The final cell activity was determined by MTT assay.

### EBV-DNA

We examined the concentration of EBV DNA in the EBV-positive ENKL cell lines, SNK-6, SNK-6/ADM and SNK-6/ADM-SP cells. First, we treated cells with histone deacetylase (HDAC) inhibitor (Epidaza) for 72 h for EBV reactivation until the virus titer was detected. The EBV DNA copy number was measured in the cell culture supernatants using quantitative PCR.

### Statistical analysis

All the experiments were performed in triplicate. All the data were analyzed with SPSS 17.0 software and expressed as mean ± standard deviation (SD). Statistical significance of differences was determined by Student’s *t*-test. A *P* value of less than 0.05 was considered significant.

## Results

### SP cells exist in SNK-6/ADM cell line

**Fig. 1:**
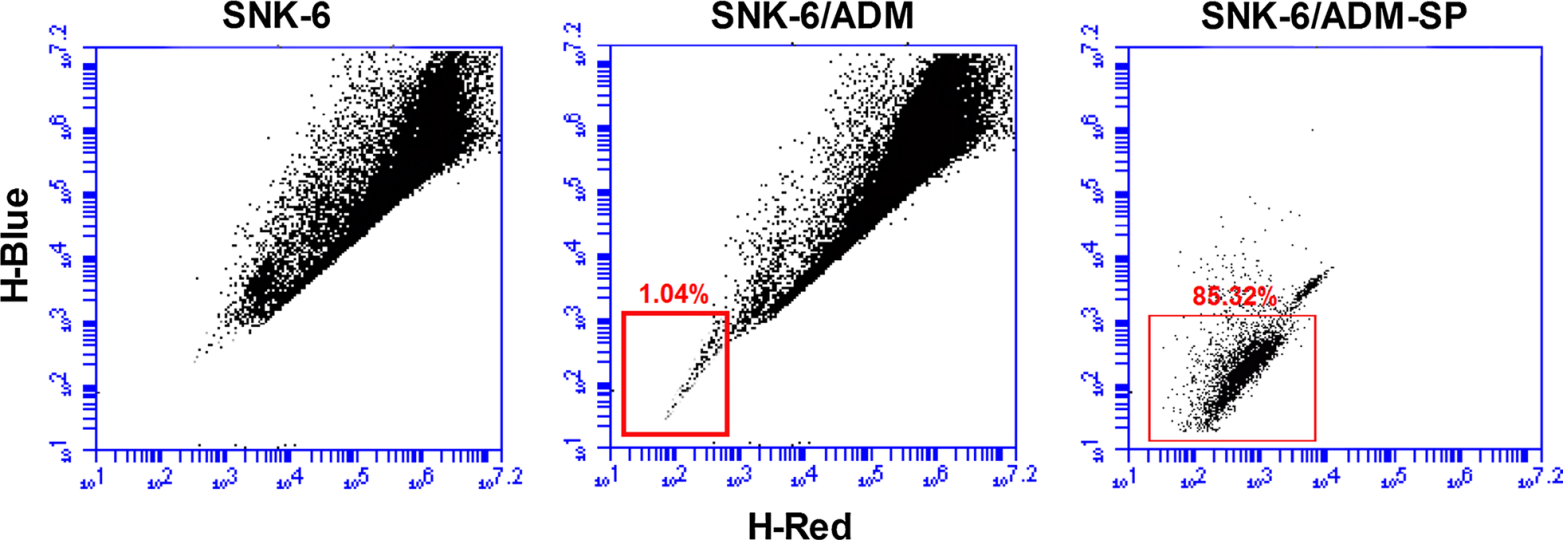
Side population cells in SNK-6/ADM cell line were detected by flow cytometry. Side population (SP) discrimination assay was performed in SNK-6 and SNK-6/ADM cells. Hoechst side population (gated) ratio in SNK-6/ADM was 1.04%. SNK-6/ADM-SP cells were sorted to 85.32% purity. However, no SP-like cells were sorted to in the SNK cells

We previously developed a doxorubicin-resistant ENKL cell line designated as SNK-6/ADM. The IC50 of SNK-6/ADM was 31.06 ± 0.27 μg/mL, compared with 6.92 ± 0.41 μg/mL of SNK-6 (Table 1). An RI of nearly 4.49 suggested increased doxorubicin resistance. Results showed SP-like cells could hardly be detected in the SNK cells. However, the SP cells with 85.32% purity ranged from 1.0 to 2.0% approximately were sorted from SNK-6/ADM cells (Fig. 1). We enriched SNK-6/ADM-SP cells for further study.

### Stronger multidrug resistance of SNK-6/ADM-SP

**Fig. 2:**
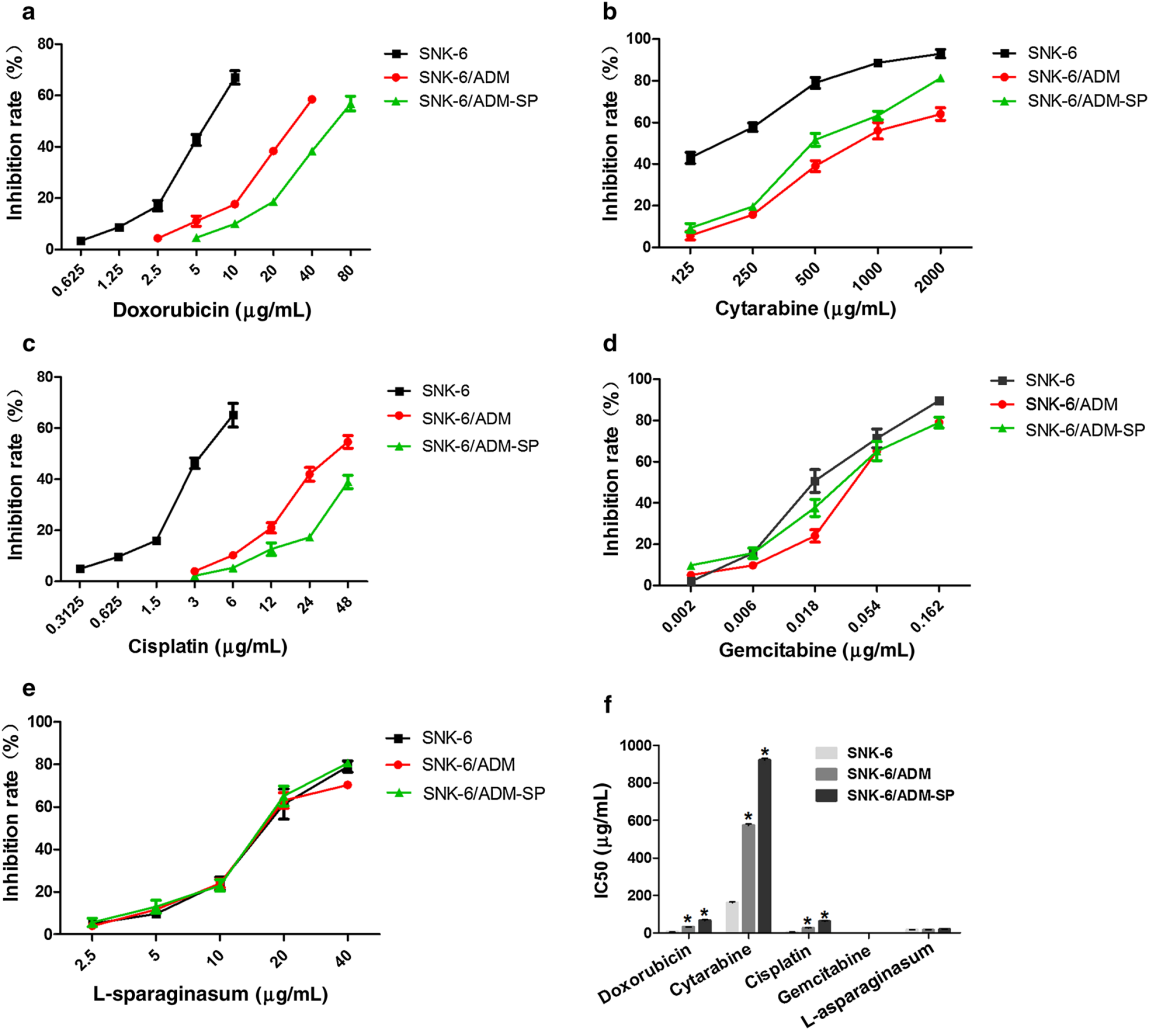
The IC50s of SNK-6, SNK-6/ADM and SNK-6/ADM-SP cells were separately treated with the five well-known chemotherapy drugs. **a**–**e** The drug response curves to various drugs and drug concentrations. **f** The upward trend of IC50s among the three cell lines is obvious in the groups of doxorubicin, cytarabine and cisplatin, but the difference almost can be neglected in the group of gemcitabine and l-asparaginasum. *P < 0.05 compared with SNK-6 cells

We further tested the multidrug resistance of SNK-6/ADM-SP for five well-known chemotherapy drugs in ENKL: doxorubicin, cytarabine, cisplatin, gemcitabine, l-asparaginasum. In order to accurately calculated IC50, we groped for the appropriate concentration gradient in the prior period, calculated the inhibition ratio of different concentration of drugs on cells (Fig. 2a–e), and finally drew a conclusion of IC50s of SNK-6, SNK-6/ADM, SNK-6/ADM-SP cells. As shown in Table 1 and Fig. 2f, SNK-6 cells were less-sensitive to cytarabine compared with other drugs with relatively high IC50 of 161.31 ± 4.04, moderate sensitive to doxorubicin, cisplatin and l-asparaginasum with IC50s of 6.92 ± 0.41, 3.71 ± 0.64 and 17.45 ± 1.04, high-sensitive to gemcitabine with IC50 of 0.02 ± 0.00. However, the IC50s to doxorubicin, cytarabine and cisplatin displayed the significantly trend of rising in SNK-6, SNK-6/ADM, and SNK-6/ADM-SP cells successively, and the corresponding RI _(SNK-6-ADM/SNK-6)_ and RI _(SNK-6-ADM-SP/SNK-6)_ to these three drugs were 4.49 and 9.65, 3.56 and 5.71, 7.09 and 16.61 respectively. By contrast, IC50s of gemcitabine and l-asparaginasum remained unchanged among three cell lines. These results indicated that SNK-6/ADM-SP presents a stronger multidrug resistance, and gemcitabine and l-asparaginasum had more potential to overcome this predicament.

**Table 1:** IC50s of 3 cell lines treated with 5 different drugs (μg/mL)

	SNK-6	SNK-6/ADM	SNK-6/ADM-SP
Doxorubicin	6.92 ± 0.41	31.06 ± 0.27*	66.75 ± 4.12*
Cytarabine	161.31 ± 4.04	574.22 ± 8.18*	921.06 ± 9.97*
Cisplatin	3.71 ± 0.64	26.32 ± 1.69*	61.64 ± 3.04*
Gemcitabine	0.02 ± 0.00	0.04 ± 0.01	0.03 ± 0.01
l-asparaginasum	17.45 ± 1.04	16.32 ± 1.39	18.96 ± 1.44

The unit of l-asparaginasum here was converted to μg/mL according to the formula (170U = 1 mg). *P < 0.05 compared with SNK-6 cells

### Morphology and proliferation of cell lines

**Fig. 3:**
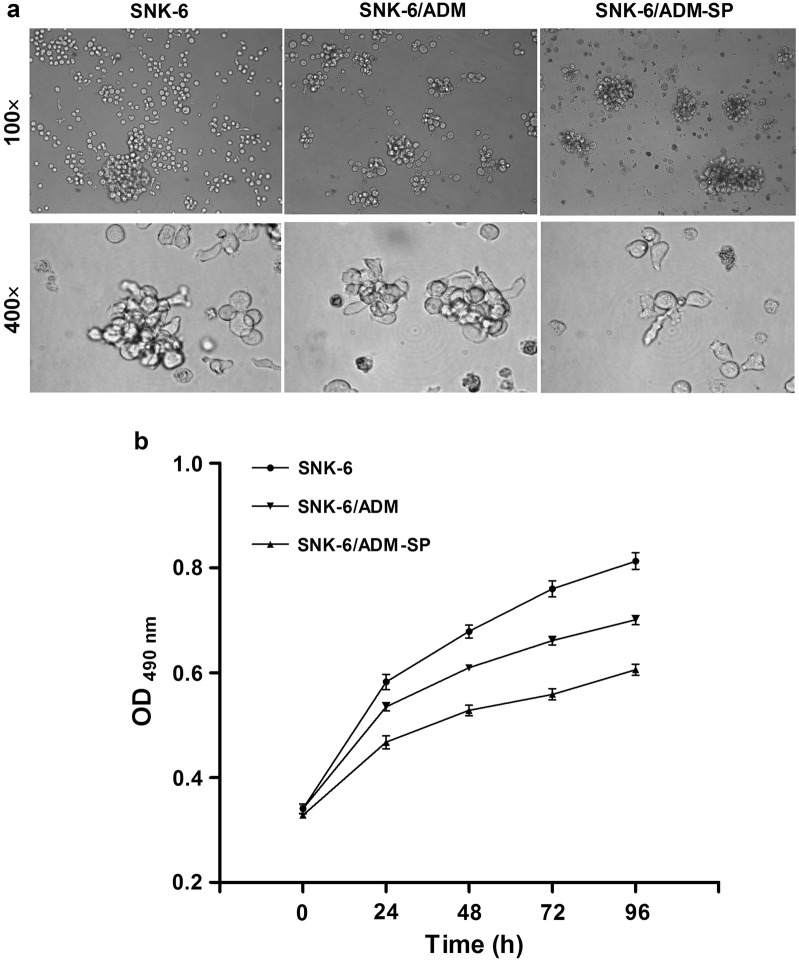
Morphology and proliferation of SNK-6, SNK-6/ADM and SNK-6/ADM-SP cells. **a** The morphology of SNK-6, SNK-6/ADM cells and SNK-6/ADM-SP cells. Compared to SNK-6 and SNK-6/ADM cells, cytoplasm of SNK-6/ADM-SP cells appeared pycnotic with scattered growth density. **b** SNK-6/ADM-SP cells demonstrated weaker cell proliferation than the other two cell lines in terms of growth curve

The morphology of SNK-6, SNK-6/ADM and SNK-6/ADM-SP cells was evaluated, and the cell number was counted to determine the proliferation capacity. Results showed that the cytoplasm of SP cells appeared pycnotic with scattered growth density (Fig. 3a). In addition, SP cells exhibited relatively poor cellular growth, with scattered growth density and slow growth rate (Fig. 3a). The SNK-6/ADM-SP cells demonstrated weaker cell proliferation than the other two cell lines in terms of growth curve (Fig. 3b).

### Expression of ATP-binding cassette (ABC) transporters

**Fig. 4:**
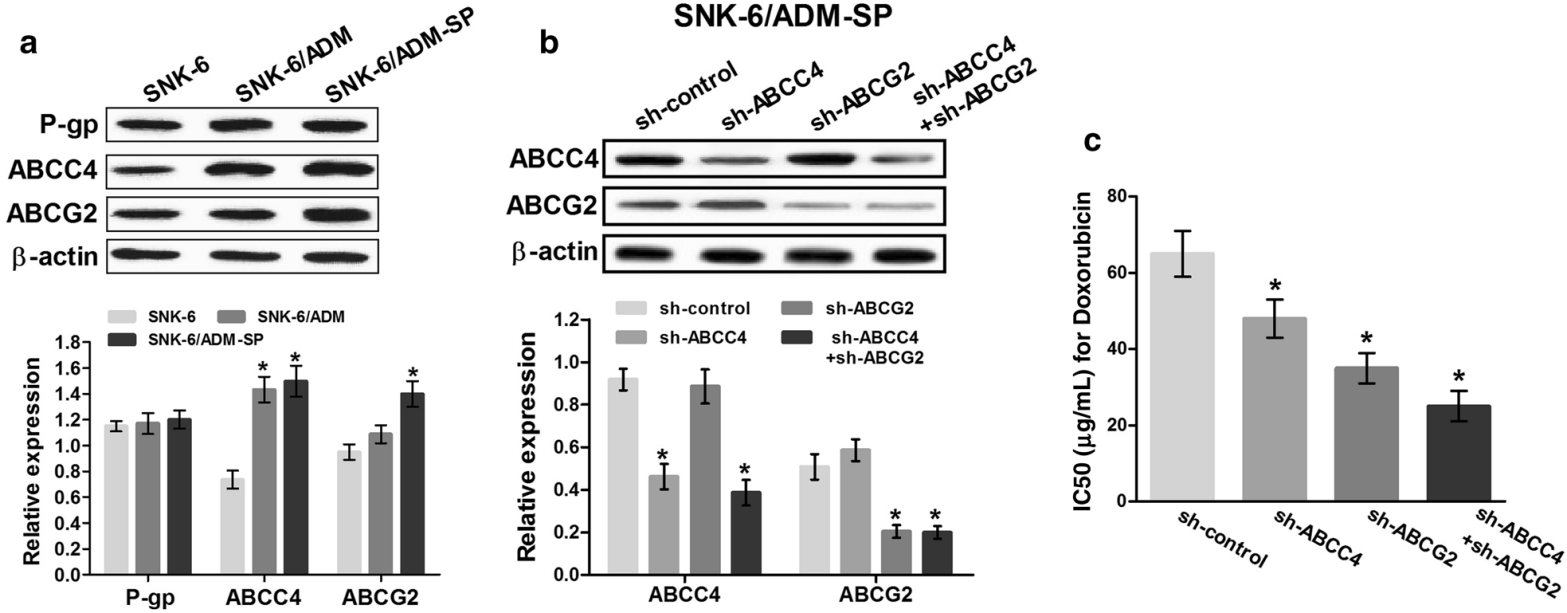
Expression of ATP-binding cassette (ABC) transporters and the effect of ABCC4 and ABCG2 knockdown on drug resistance in SNK-6/ADM-SP cells. **a** Expression of ABC transporters was examined in SNK-6, SNK-6/ADM and sorted SNK-6/ADM-SP cells. ABCC4, rather than ABCB1 (P-gp) protein was expressed higher in SNK-6/ADM or SNK-6/ADM-SP cells, while ABCG2 was expressed higher in SNK-6/ADM-SP cells. **b** The expression of ABCC4 and ABCG2 in SNK-6/ADM-SP cells treated with short-hairpin RNA plasmids directly knocking down ABCC4 (sh-ABCC4), ABCG2 (sh-ABCG2) or the non-targeting sequence (sh-control). **c** The effect of ABCC4 and ABCG2 knockdown on IC50 of doxorubicin in SNK-6/ADM-SP cells. *P < 0.05 compared with SNK-6 cells or the control group

To further validate the expression of ABC transporters possibly associated with drug resistance or efflux of SP cells in SNK-6, SNK-6/ADM and SNK-6/ADM-SP cells, the expression of ABCB1 (P-glycoprotein, P-gp), ATP-binding cassette sub-family G member 2 (ABCG2) and multidrug resistance-associated protein 4 (ABCC4) were quantitatively analyzed. We utilized western blot to examine the protein expression. As shown in Fig. 4a, ABCC4, rather than ABCB1 protein was expressed higher in SNK-6/ADM or SNK-6/ADM-SP cells, while ABCG2 was significantly elevated in SNK-6/ADM-SP cells. Then to further investigate the effect of ABCC4 and ABCG2 on IC50 of doxorubicin in SNK-6/ADM-SP, the short-hairpin RNA plasmids directly knocking down ABCC4 (sh-ABCC4), ABCG2 (sh-ABCG2), sh-ABCC4 combined with sh-ABCG2, or its non-targeting sequence (sh-control) were transfected into SNK-6/ADM-SP cells (Fig. 4b). The results demonstrated that the IC50 of doxorubicin was significantly decreased in sh-ABCC4-transfected, sh-ABCG2-transfected and sh-ABCC4 + sh-ABCG2-transfected cells compared with the sh-control groups (Fig. 4c). On the other hand, this phenomenon was more obvious in cells transfected with sh-ABCC4 combined with sh-ABCG2. These results suggested that doxorubicin resistance of SNK-6/ADM and SNK-6/ADM-SP cells were probably dependent on ABCC4 expression, and the conservative properties of SNK-6/ADM-SP cells were ABCG2-dependent.

### Cell cycles of SNK-6, SNK-6/ADM and SNK-6/ADM-SP

**Fig. 5:**
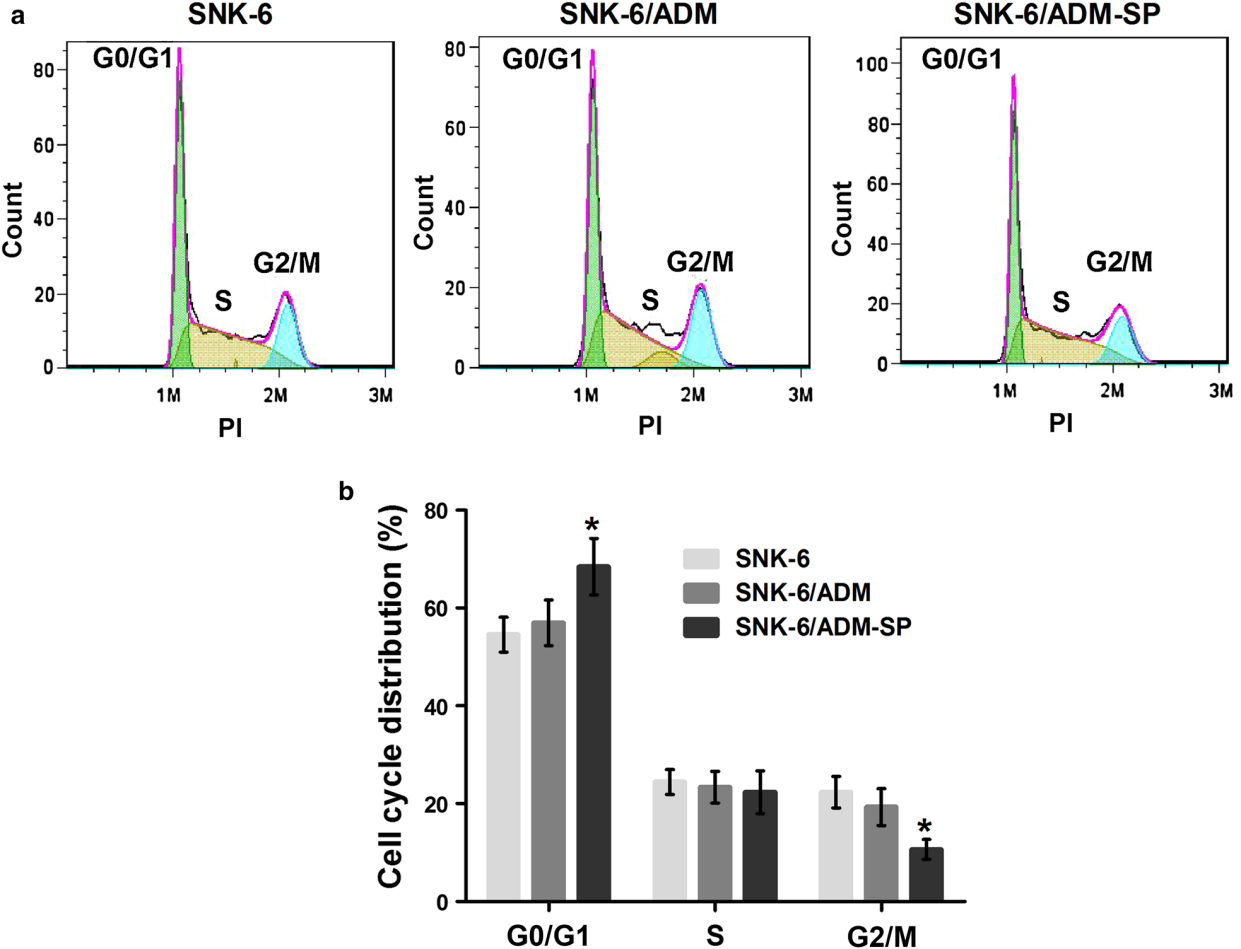
Cell cycles of SNK-6, SNK-6/ADM and SNK-6/ADM-SP. **a** Cell cycles were determined by flow cytometry. **b** The statistics of each cell cycle phase. *P < 0.05 compared with SNK-6 cells

Flow cytometry analysis showed that the proportion of SNK-6/ADM-SP cells in G0/G1 phase was higher than that in SNK-6 (*P* < 0.05), and SNK-6/ADM cells, suggesting that most SNK-6/ADM-SP cells remained at stationary stage (Fig. 5a, b).

### Expression of surface markers

**Fig. 6:**
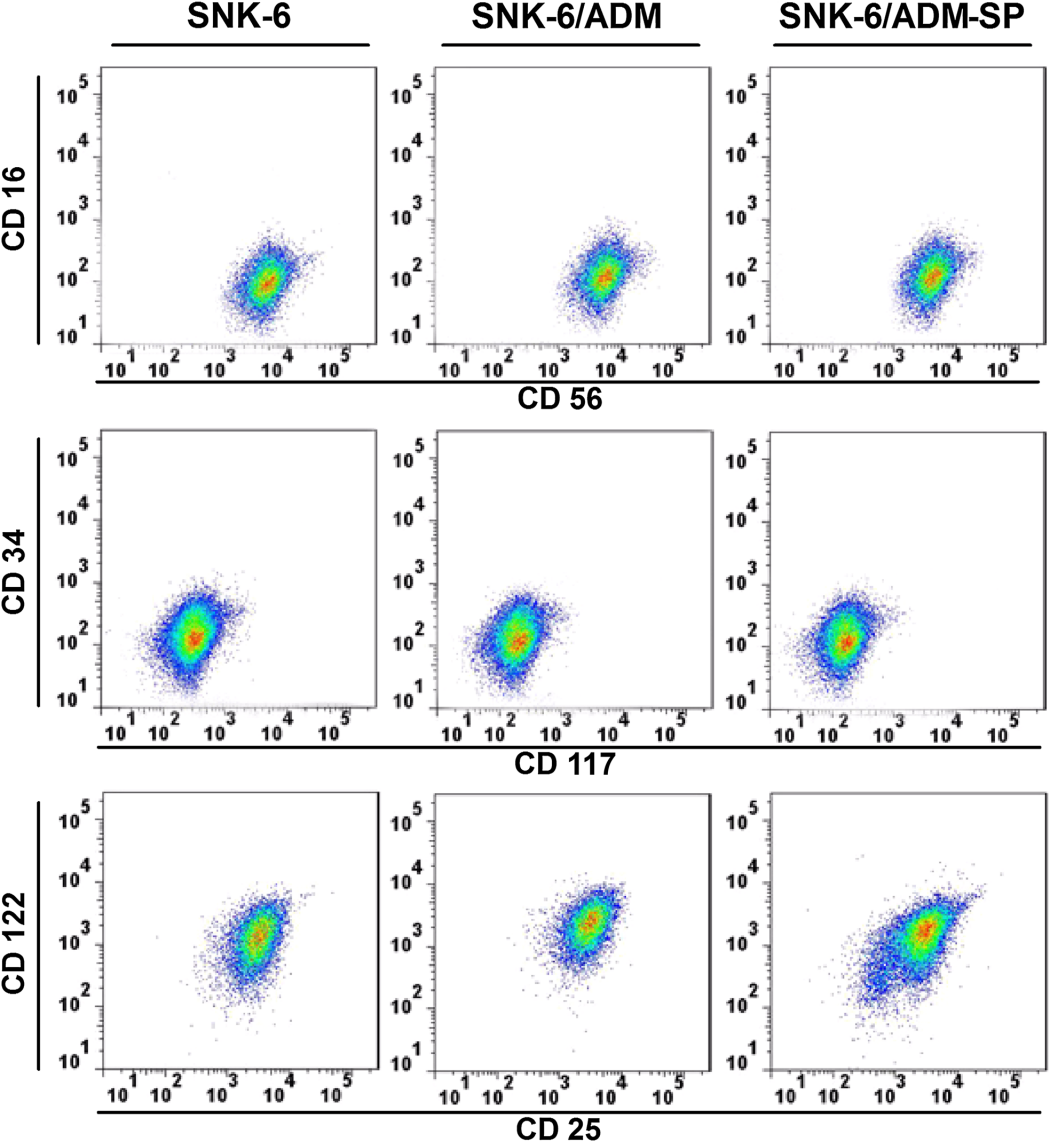
Expression of surface markers. CD56, CD16, CD34, CD117, CD25 (IL-2 receptor ɑ) and CD122 (IL-2/15R-β) were detected by flow cytometry. Three cell lines were similarly CD56+, CD16−, CD34−, and CD117−, suggesting that SNK-6/ADM-SP was still a mature NK cell-derived cell line. The expression of CD25 and CD122, which assess the developmental potential of lymphocyte was decreased in SNK-6/ADM-SP

Previous studies confirmed that SNK-6 cells were CD3−CD4−CD8−CD16−CD19−CD21−CD25+CD56+CD57+HLA−DR+, and exhibited NK-cell phenotype. In this study we identified CD56, CD16, CD34, and CD117 to determine NK-cell phenotype and maturity of SNK-6, SNK-6/ADM and SNK-6/ADM-SP cells, and CD25 (IL-2 receptor ɑ) and CD122 (IL-2/15R-β) to assess the developmental potential. Results showed that the expression of CD56+, CD16−, CD34−, and CD117− cells was similar in the three cell lines, suggesting that SNK-6/ADM-SP was still a mature NK cell-derived cell line. However, the expression of CD25 and CD122 was decreased in SNK-6/ADM-SP, suggesting potential different response to cytokines like IL-2 and IL-15 in these cells (Fig. 6).

### IL-15-sensitivity and EBV-inhibition of SNK-6/ADM-SP cells

**Fig. 7:**
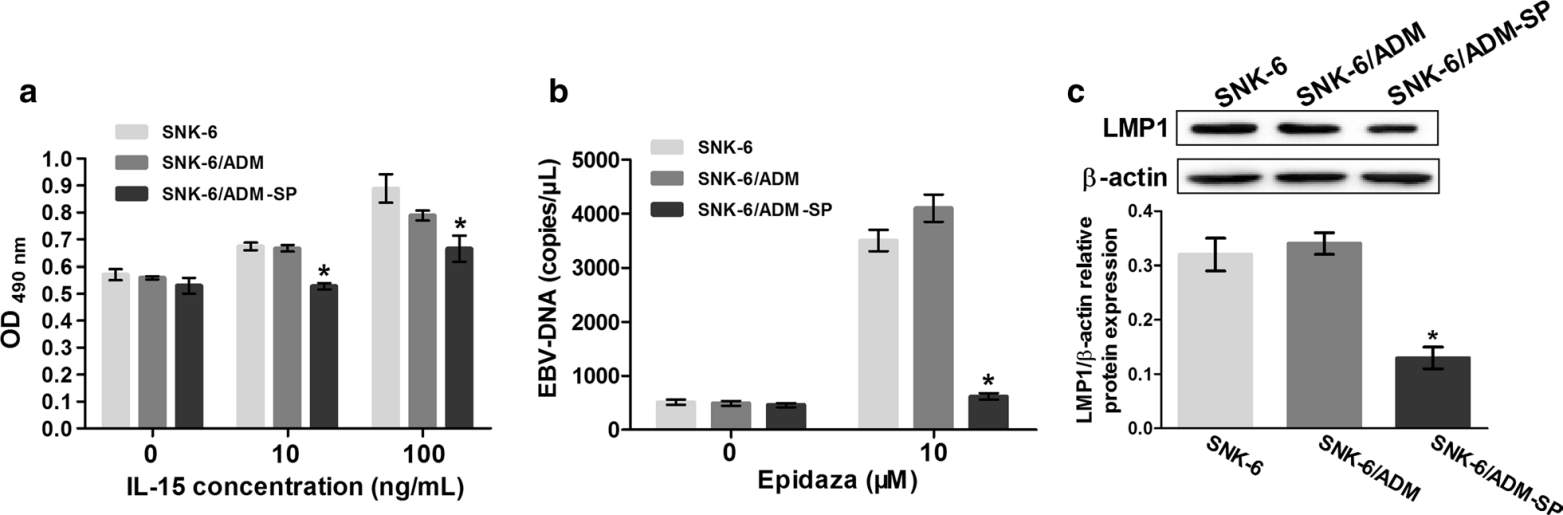
IL-15-sensitivity and EBV-inhibition of SNK-6/ADM-SP cells. **a** MTT assay revealed that IL-15 stimulated cell reproduction, and enhanced proliferation. However, this ability was decreased in SNK-6/ADM-SP cells. **b** EBV-DNA copies were detected at  3.0 − 4.5 × 10^3^ copies/μL in SNK-6 and SNK-6/ADM after treatment with HDAC inhibitor Epidaza, but it was difficult to quantify in SNK-6/ADM-SP cells. **c** The expression of EBV-major protein LMP1 in cells without HDAC inhibitor was decreased in SNK-6/ADM-SP cells.*P < 0.05 compared with SNK-6 cells

SNK-6, SNK-6/ADM and SNK-6/ADM-SP cells (4 × 10^4^ per well) were treated with 0, 10, 100 ng/mL of IL-15 for 48 h. MTT assay revealed that IL-15 stimulated cell reproduction, and enhanced proliferation. However, this ability was decreased in SNK-6/ADM-SP cells due to decreased expression of CD122 potentially (Fig. 7a).

Routine examination was not sufficient to quantify EBV-DNA other than viral activation. After treatment with HDAC inhibitor, EBV-DNA copies were detected at 3.0 − 4.5 × 10^3^ copies/μL in SNK-6 and SNK-6/ADM. However, they were still difficult to quantify in SNK-6/ADM-SP cells (Fig. 7b). We also analyzed the expression of EBV-major protein LMP1 in cells without HDAC inhibitor by western blot. Results showed that the expression of LMP1 was decreased in SNK-6/ADM-SP cells (Fig. 7c).

## Discussion

ENKL is a distinct clinicopathological entity and EBV-associated disease that is highly aggressive, with a geographic predilection for Asia, Central and South America [12, 13]. Current treatment strategies are not effective and chemoresistance leads to poor prognosis [5, 14]. The mechanism of oncogenesis and biological characteristics of ENKL which provide important insights into potential therapeutic options is not clear [4].

The origin and differentiation of lymphatic leukemia and B cell lymphomas are well described. Previous studies have suggested that oncogenesis of hematological malignancy occurs via two principal mechanisms: transformation of hematopoietic stem cells and progenitor cells committed to tumor-initiating cells, and differentiation along the original lineage. Most lymphomas are more likely to originate from normal lymphocytes during specific developmental stages [15–17]. However, the oncogenic features of ENKL lymphomas and even normal NK cells are less clear. The current hypothesis is that traditional NK cells originate in the bone marrow from a common T cell/NK cell progenitor, and develop to maturity [18, 19]. Nevertheless, the NK cell phenotype and function in mucous and other extranodal tissues vary from that of traditional NK cells [20]. The origin and relationship with local microenvironment remain to be elucidated. Thus, ENKL cells manifest distinct biology and clinicopathology that distinguish them from other T cell or NK cell-derived hematological malignancies. Perhaps, this cellular heterogeneity is a major factor underlying the unique biological characteristics and elusive response to chemotherapy of ENKL.

In the previous study, we developed a doxorubicin-resistant ENKL cell line known as SNK-6/ADM based on classical ENKL cell line SNK-6 [CD3ε+, CD20−, CD56+, CD3(Leu4)−, EBER(+)] [3]. We originally scheduled to sort the SP cells to investigate the oncogenesis of ENKL. However, we could hardly found SP cells among normal SNK-6 cells. Fortunately, SP cells varied from approximately 1% to 2% of SNK-6/ADM cells were successfully enriched and designated as SNK-6/ADM-SP. Due to the abundance of studies reporting SNK-6 phenotype, we identified the origin of these SP cells by evaluating CD56, CD16, CD34 and CD117 expression. Results showed that SNK-6/ADM-SP cells originated from mature NK cells, which were similar to normal SNK-6 and SNK-6/ADM cells. We further evaluated the sensitivity of chemotherapy drugs of three cell lines, and results showed that ENKL cells were originally not sensitive to traditional chemotherapy drugs doxorubicin and cytarabine with higher IC50s. Furthermore, the resistence indexes of doxorubicin, cytarabine and cisplatin in SNK-6/ADM and SNK-6/ADM-SP cells were significantly increased, especially in the SNK-6/ADM-SP cells. That means SNK-6/ADM-SP cells have stronger multidrug resistance even than doxorubicin-resistant ENKL cell line. In contrast, IC50s of gemcitabine and l-asparaginasum among three cell lines were had almost no change. Moreover, gemcitabine also showed a wonderful sensitivity to normal or resistant ENKL cells. These results were exactly consistent with our latest advances of clinical trial [5, 6].

Consequently, we developed some further studies in order to explore the biological characteristics and mechanism of drug-resistance of SNK-6/ADM-SP cells. Morphological and proliferation assays showed that SNK-6/ADM-SP cells manifested indolent status, mostly were restricted to stationary phase. The control SNK-6 cells showed effective proliferation. These preliminary observations suggested that SNK-6/ADM-SP possess distinct biological characteristics as independent cell populations. SP cells were separated from many tissues including malignant tumors by sorting cancer stem cells [11]. One study [21] found that a low concentration of doxorubicin increased the proportion of SPs in acute leukemia cell line HL60. Further studies [22] demonstrated that the efflux of Hoechst 33342 by SP cells was mainly mediated by ATP-binding cassette sub-family G member 2 (ABCG2), one of the ATP-binding cassette (ABC)-type transporters. They displayed stem cells-like features. However, a few other studies indicated that SP cells might be a subgroup of heterologous cell populations with ‘SP phenotype’, with stem cell characteristics, multidrug resistance or other potential [23, 24]. In our study, we did not consider SNK-6/ADM-SP as cancer stem-like cells because of their mature NK cell-derived phenotype and indolent cellular characteristics but as potentially super multidrug resistant cells. Therefore, the expression of ABCB1, ABCG2 and ABCC4 was detected in SNK-6, SNK-6/ADM, and SNK-6/ADM-SP cells. The results showed that the expression of ABCC4 in SNK-6/ADM and SNK-6/ADM-SP were both higher than that in SNK-6, while the ABCG2 expression was significantly increased in SNK-6/ADM-SP cells. What was more, we considered that the main factor underlying SNK-6/ADM drug resistance was ABCC4 and ABCG2 rather than P-gp, which is the most classic MDR protein, and consistent with recent studies [4, 25] suggesting the absence of absolute correlation between P-gp and chemotherapy resistance. Further results showed that the doxorubicin resistance in SNK-6/ADM-SP cells transfected with shRNAs targeting ABCC4, ABCG2 and ABCC4 + ABCG2 was significantly decreased compared with the sh-control groups, indicating the vital roles of ABCC4 in drug resistance and ABCG2 in conservative characteristics of SNK-6/ADM-SP cells.

Previous studies elucidating the process of normal NK-cell development provide comparative support for investigations into the differentiation patterns of ENKL [26, 27]. Studies [28, 29] reported that IL-15 plays an important role in the survival of human natural killer (NK) cells. In human bone marrow and secondary lymphoid tissue (SLT), CD34+ hematopoietic stem cells and hematopoietic progenitor cells differentiate into cytolytic NK cells after stimulation with either IL-2 or IL-15 [30]. We investigated the role of IL-15 in the proliferation of three cell lines SNK-6, SNK-6/ADM, SNK-6/ADM-SP. Cells cultured in a medium containing IL-2 over several generations were not evaluated for IL-2. Based on our analysis, we found that SNK-6/ADM-SP showed an obvious reduction in sensitivity to IL-15. Detection of CD122 (IL-2/15R-β) showed that downregulation of expression resulted in poor sensitivity of SNK-6/ADM-SP cells to IL-15. These phenomena also suggested that SNK-6/ADM-SP cells might be a subgroup of indolent or conservative population of cells.

Nasal ENKL is characterized by EBV infection. SNK-6 cell line is representative of EBV-positive ENKL cell origin. EBV infects more than 90% of the population and mostly exhibits latency following infection. However, EBV is reactivated from the latent phase to the lytic phase and produces infectious viral progeny under changing external conditions. A recent study has revealed that the HDAC inhibitor could reactivate EBV in SNK-6 cell line [31]. In our study, SNK-6 cells and other cell lines were not analyzed for EBV DNA possibly because of the latent infection leading to extremely low virus load. After treatment with HDAC inhibitor (Epidaza), we found EBV-DNA (approximately 3.0 − 4.5 × 10^3^ copies/μL) in SNK-6 and SNK-6/ADM, but rarely in SNK-6/ADM-SP cells. We speculated that the decreased EBV DNA was associated with the conservative characteristics of SNK-6/ADM-SP cells. The following analysis of LMP1 expression suggested that EBV-related protein decreased in SNK-6/ADM-SP cells, further suggesting that SNK-6/ADM-SP cells exhibited a conservative virus inhibition. Maybe self-protection mechanism in the evolution process of lymphocyte was involved in this phenomenon.

## Conclusion

We identified the presence of SP-like subgroup of cells in doxorubicin-resistant ENKL cell line, characterized by stronger multidrug resistance and conserved performances including relatively poor proliferation, weak sensitivity to cytokines, and EBV inhibition. Based on our findings, we believe that ENKL cells comprise heterogeneous populations rather than a uniform clone. This cellular heterogeneity is a major factor underlying the unique biological characteristics and elusive response to chemotherapy of ENKL. We will conduct a series of cytogenetic and immunological studies of this subgroup of cells and diverse samples, to elucidate this intractable disease.
